# Diaphragmatic crural eventration

**DOI:** 10.4103/0971-9261.42568

**Published:** 2008

**Authors:** K. Sivakumar

**Affiliations:** Department of Pediatric Surgery, SAT Hospital, Medical College, Trivandrum; Medical College, Kottayam, India

**Keywords:** Diaphragmatic defects, gastric volvulus, right crus of diaphragm

## Abstract

**Aim:**

We evaluated patients with gastric volvulus secondary to diaphragmatic pathology.

**Materials and Methods:**

Eight patients (5 males and 3 females) presented to the author in a tertiary care center during 1997-2006 were analyzed in terms of age, sex, symptomatology, diagnosis and predisposing factors.

**Observations:**

Six had an acute presentation and rest had chronic symptomatology. The two patients who had total gangrene stomach died postoperatively and one patient died preoperatively due to aspiration. All the cases presented with acute symptoms had diaphragmatic pathology, and out of these, three cases had the specific entity, which is named as diaphragmatic crural eventration.

**Conclusions:**

Diaphragmatic crural eventration is characterized by the defective development of the right crus of diaphragm, and this is embryologically significant as the right crus and ligaments of the stomach develop from dorsal mesoesophagus and mesogastrium. The author recommends a closer look for this defect of diaphragm while operating a case of gastric volvulus.

## INTRODUCTION

Gastric volvulus is relatively rare in children. The incidence peaks in those in the age group of 40-50 years. Approximately, 20% occur in children and of this majority occur in infancy.[1] When one encounters a case of gastric volvulus, he/she should always look for associated the pathology of stomach, gastric ligaments, spleen, colon and for defects in diaphragm.[2] Two thirds of the cases are associated with diaphragmatic pathology.[1] The usual diaphragmatic pathologies are eventration diaphragm, congenital diaphragmatic hernia, rupture of diaphragm, hiatus hernia[2] Morgagni's hernia[3] and to add one more type of diaphragmatic defect, the diaphragmatic crural eventration, through this paper.[4,5]

The objective of this study is to identity the diaphragmatic pathology observed in cases of gastric volvulus and to highlight the new terminology “diaphragmatic crural eventration” and its embryo pathogenic correlation with the gastric volvulus.

## MATERIALS AND METHODS

The cases of gastric volvulus the author had personally encountered during the period of 1997 to 2006 were preoperatively and peroperatively assessed and analyzed in terms of age, sex, clinical features, type of gastric volvulus and the associated pathology of diaphragm and ligaments of stomach.

## RESULTS

There were eight cases of gastric volvulus during this period. Male: Female ratio was 5:3. Five patients were infants. The mean age of presentation was 24.6 months (The youngest one was 14 days of age and oldest one was 9 years of age).

### 

#### Clinical features:

The presentation was with acute symptoms in six cases. The acute symptoms and signs observed were as follows: epigastric pain, vomiting, retching, epigastric fullness with mass, respiratory distress, pneumoperitoneum, bleeding per rectally and shock. In rest of the two cases, the presentation was with chronic symptoms such as intermittent cry or irritability, recurrent non-bilious vomiting and failure to thrive.

#### Investigations:

Acute cases were diagnosed by plain X-ray abdomen. A distended stomach with fluid level associated with diaphragmatic pathology aroused the suspicion of volvulus and then confirmed by upper GI contrast study. Chronic cases were diagnosed by upper GI contrast study when done as part of the evaluation of recurrent non-bilious vomiting. None of them had CT scan or other imaging techniques.

#### Operative findings:

All the patients were operated except one patient who died preoperatively due to aspiration pneumonitis. This patient had chronic presentation as the failure to thrive. (Birth weight was 3.5 kg, and at 2 months of age, the weight was 2.3 kg.)

##### Type of Gastric volvulus:

It is determined by analyzing mainly the contrast pictures and peroperative findings. Organoaxial type volvulus is observed in six cases and mesenteroaxial type is observed in one case and not defined in one case. *Stomach*: It was gangrenous in two cases and both of these patients died postoperatively. The gastric ligaments were very much attenuated in five cases and absent in two cases. *Diaphragmatic pathology*: In all the six cases with acute presentation, there was associated diaphragmatic pathology. The diaphragm was normal in the two cases with chronic presentation. Classical left hemidiaphragm eventration was observed in two cases. Late-presenting congenital diaphragmatic hernia was observed in another case. In the rest of the three cases, the right crus of diaphragm were absent.

##### Defect of right crus of diaphragm (diaphragmatic crural eventration):

This was noted in three patients. (age: 8 months, 1½ years and 4½ years; male/female ratio - 1:2.) All of them presented with acute symptoms.

### Case 1

**Figure 1:**
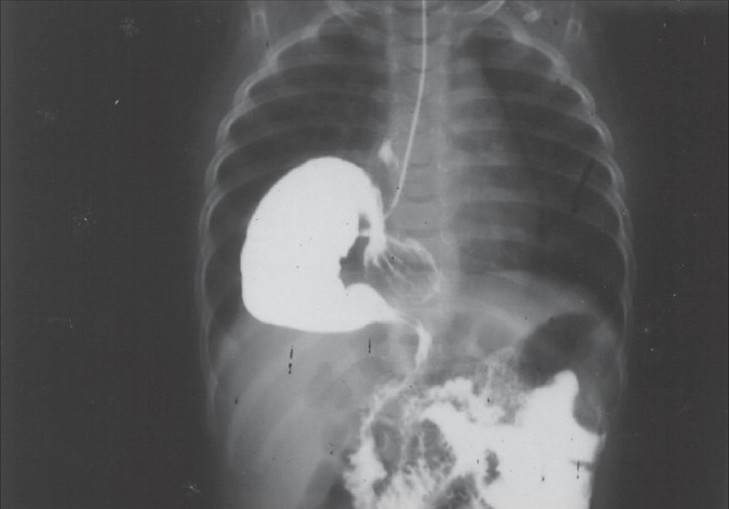
Case 1 - Barium meal picture showing organoaxial volvulus

A 2-month-old female child presented with organoaxial volvulus. The greater curvature of the entire stomach found twisted upwards and anteriorly so as to reach a level above the cardioesophageal junction [Figure 1]. The twisted stomach was within the sac formed by the attenuated right crus of diaphragm, which in turn protruded to the right hemi thorax, giving a picture of the partial eventration of the diaphragm in the X-ray. Left crus developed well. Stomach ligaments were absent. The triangular ligaments of the left lobe of the liver were divided to enable the retraction of liver and the defect of right crus was repaired by plicating the attenuated part with 2-0 prolene. The left lateral bites started from the left crus of the diaphragm. The esophageal hiatus is recreated, leaving one finger loose space for the esophagus. Gastropexy was performed by fixing the greater curvature of stomach from the level of fundus to the left dome of the diaphragm and parietal peritoneum. Postoperatively, the patient developed persistent non-bilious vomiting. Barium meal study was performed, which revealed stasis in the stomach. Hence, re laparotomy and pyloroplasty were done. The child became asymptomatic thereafter. The child is 5 years now and doing well.

### Case 2

**Figure 2:**
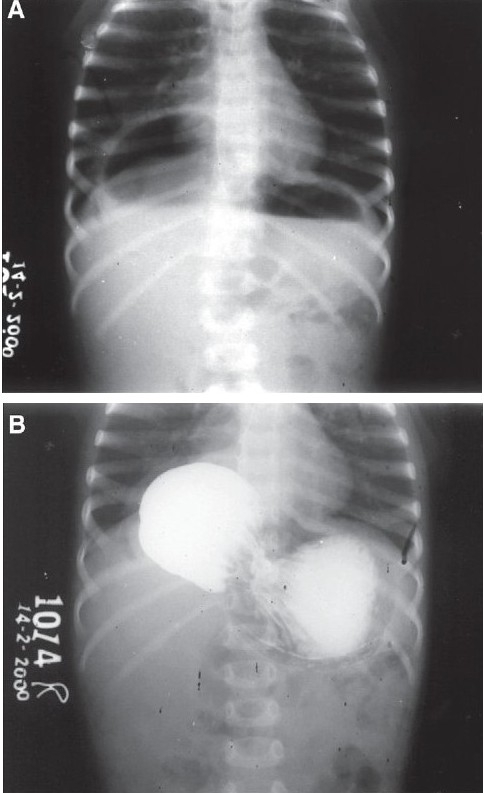
Case 2 (A) Plain X-ray showing right partial eventration (crural eventration). (B) Barium meal picture: mesenteroaxial volvulus

This case was a 1½-years-old male child. Here, combined type of volvulus with mainly mesenteroaxial orientation was found. The fundus of stomach located below the left dome of diaphragm and rest of stomach twisted anteriorly so that the antrum and pylorus are found to occupy a sac situated towards right hemi thorax [Figures 2A and B]. The right crus of diaphragm was absent and was replaced by a thin stretched-out sac. This resembled the partial eventration of diaphragm in plain X-ray. Stomach and splenic ligaments were very lax and thin. The left lobe of liver was retracted even without dividing the triangular ligaments and the defect was repaired with 2-0 prolene as described in Case 1. It was not certain how far the pericardium was adherent on to the thoracic side of the defect; however, there was no problem peroperatively and postoperatively. Gastropexy was also performed. The child did well after the surgery. There were no specific problems during the follow-up.

### Case 3

**Figure 3:**
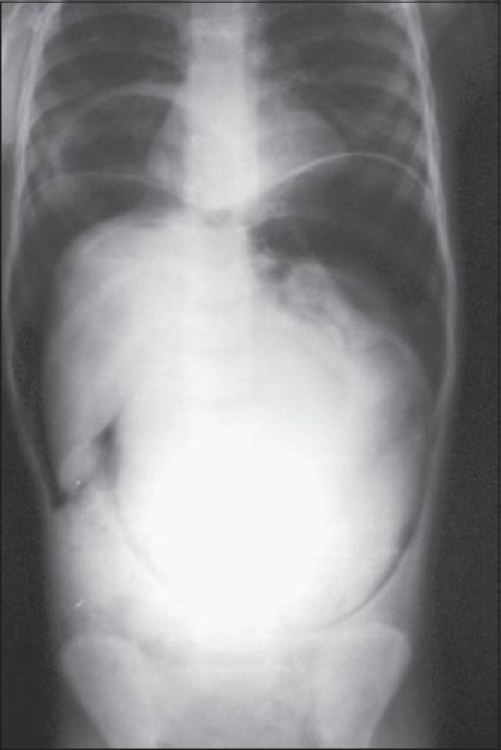
Case 3 - Pneumoperitoneum with central stomach shadow and diaphragmatic defect (crural eventration)

A 4½ yrs old female presented with tense abdominal distension, bleeding per rectally with severe pallor and shock. Plain X-ray abdomen revealed pneumoperitoneum, right-sided partial diaphragmatic eventration (actually the eventration of right crus of diaphragm) and a central abdominal shadow, which was suggestive of stomach with contents [Figure 3]. Emergency laparotomy revealed the total gangrene of stomach and defect in the right crus of the diaphragm. The type of volvulus could not be made out. Gastrostomy was performed and the abdomen closed without any other procedure. Patient expired in the immediate postoperative period.

## DISCUSSION

Stomach is held in its normal position by its natural ligaments, namely, gastrohepatic, gastrophrenic, gastrosplenic and gastrocolic ligaments. Since these ligaments are related to diaphragm, spleen and colon, any problems of these ligaments as such or the adjacent diaphragm spleen or colon can produce volvulus.[2]

Gastric volvulus is defined as abnormal rotation of all or part of stomach for more than 180° that may lead to closed-loop obstruction and possible strangulation.[1] Berti first described it in 1866.[2] In 1899, Oltmann first reported the pediatric case.[6] In 1904, Borchardt described the classical triad of severe epigastric pain, retching or vomiting and inability to pass nasogastric tube.[2] However, one need not find the classical triad in all the cases.

Most of the studies show that males are affected more than females.[2,7] This was same also in this study; however, the specific entity of diaphragmatic crural eventration was found to be greater in females.

According to the axis of rotation, gastric volvulus is classified into the following: organoaxial (60%) mesenteroaxial (30%), mixed type (nearly 2%) and the rest are unclassified.[1,2] Organoaxial is more common and usually associated with diaphragmatic pathology, and there is 25% chance of strangulation. Perforation of stomach can also occur.[8]

Splenic pathology such as wandering spleen[9] or asplenia[10] may be associated with increased incidence of gastric volvulus. Although during this period we had a case of torsion of wandering spleen, he never presented with the features of gastric volvulus before splenopexy or even during the follow-up after 10 years.

In situations where diaphragmatic pathology is absent, the absence or the laxity of the ligament of stomach was found to be responsible. Dalgaard first noted this in cadaver.[11] Eventration of diaphragm is the commonest associated diaphragmatic pathology, and it is followed by congenital diaphragmatic hernia, which were well described in literature.

**Figure 4:**
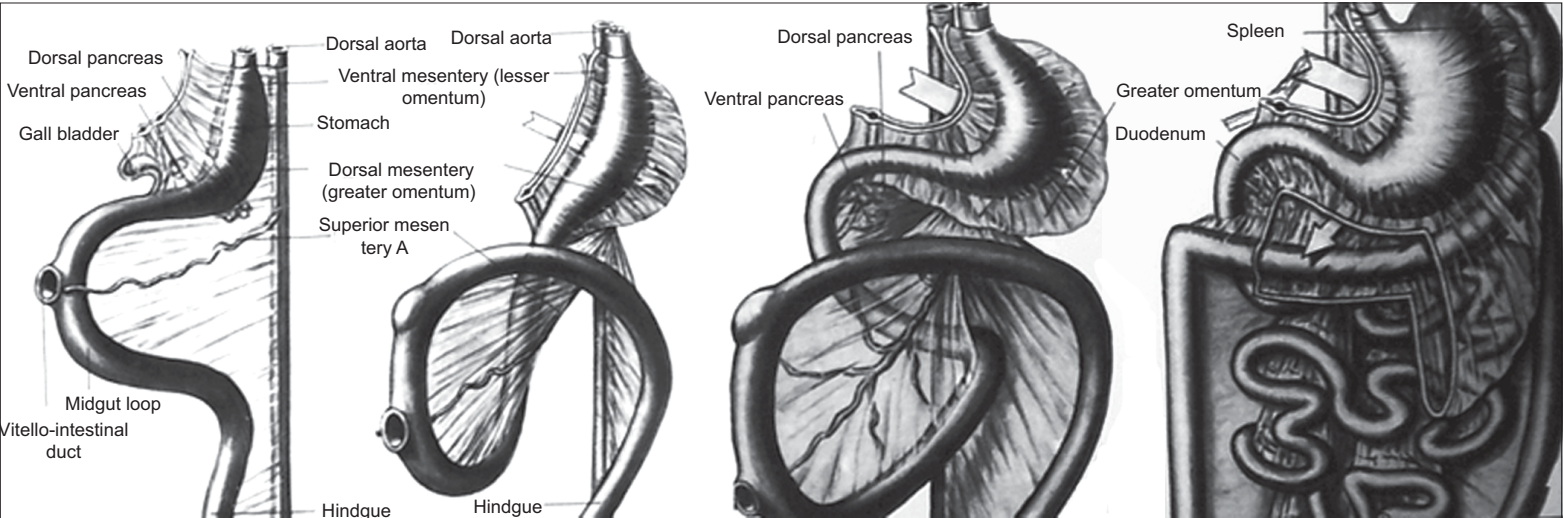
Embryology of dorsal mesoesophagus, mesogastrium and ligaments of stomach

In this study, there were three cases, where gastric volvulus was associated with attenuated right crus of diaphragm. This association has got a good embryological backup [Figure 4]. The dorsal mesoesophagus and mesogastrium play an important role in the proper development of the right crus of the diaphragm, gastrophrenic, gastrosplenic, spleenorenal, gastrocolic ligaments and lesser sac.[12] Therefore, a defect in the development of dorsal mesoesophagus and mesogastrium leads to defective right crus and gastric ligaments. The right crus will get attenuated to form a thin membrane to the right of esophageal hiatus from its level of vertebral origin and protrude to the right hemithorax, and this along with the defective gastric ligaments predisposes to the gastric volvulus. This entity can be clinically suspected when a plain X-ray chest and abdomen show right-sided partial eventration, where the partial eventration is located to the right of cardiac shadow in the anteroposterior film and posteriorly near vertebra in the right lateral film. The content is stomach and its status is better delineated by upper GI contrast study. While operating such cases, one has to plicate the defect and one must be careful about the pericardium and vagus nerve. The esophageal hiatus must be reconstructed. Pyloroplasty is not necessary, but it should be done if one damages the vagus nerve. The outcome is very good and depends on the viability of the stomach.

Although it is embryologically well-established that the right crus of the diaphragm develops from dorsal mesoesophagus, a defective right crus of diaphragm is not reported thus far as a separate entity. The chart for all the diaphragmatic defects[13] does not mention the defects of the crus of diaphragm. In situation of paraesophageal hiatus hernia, the hiatus is found to be sufficiently wide for stomach to roll or slide in; however, there is no mention of the crural pathology. Congenital paraesophageal hiatus hernia by means is not well established.[13]

The correlation of the right crus defect and gastric volvulus is important and should be grouped as a separate entity called diaphragmatic crural eventration. Paraesophageal hiatus hernia should be reserved for the acquired condition seen in adults since its congenital nature is not established. Hence, the author recommends a closer examination for this crural defect while operating a case of gastric volvulus or hiatus hernia in children.
